# Chronic Bronchitis in Children and Adults: Definitions, Pathophysiology, Prevalence, Risk Factors, and Consequences

**DOI:** 10.3390/jcm13082413

**Published:** 2024-04-20

**Authors:** Jingwen Zhang, Danielle F. Wurzel, Jennifer L. Perret, Caroline J. Lodge, E. Haydn Walters, Shyamali C. Dharmage

**Affiliations:** 1 Allergy and Lung Health Unit, Centre for Epidemiology and Biostatistics, Melbourne School of Population and Global Health, The University of Melbourne, Melbourne, VIC 3053, Australia; jingwen.zhang7@unimelb.edu.au (J.Z.); danielle.wurzel@unimelb.edu.au (D.F.W.); jennifer.perret@unimelb.edu.au (J.L.P.); clodge@unimelb.edu.au (C.J.L.); haydn.walters@utas.edu.au (E.H.W.); 2 Murdoch Children’s Research Institute, The Royal Children’s Hospital, Melbourne, VIC 3052, Australia; 3 Institute for Breathing and Sleep (IBAS), Melbourne, VIC 3084, Australia; 4 School of Medicine, University of Tasmania, Hobart, TAS 7000, Australia

**Keywords:** chronic bronchitis, chronic cough, protracted bacterial bronchitis

## Abstract

The complex nature of chronic bronchitis (CB) and changing definitions have contributed to challenges in understanding its aetiology and burden. In children, CB is characterised by persistent airway inflammation often linked to bacterial infections and is therefore termed “protracted bacterial bronchitis” (PBB). Longitudinal studies suggest that CB in childhood persists into adulthood in a subgroup. It can also be associated with future chronic respiratory diseases including asthma, bronchiectasis, and chronic obstructive pulmonary disease (COPD). Adult CB is traditionally associated with smoking, occupational exposures, and lower socioeconomic status. The interplay between risk factors, childhood CB, adult CB, and other chronic respiratory diseases is intricate, requiring comprehensive longitudinal studies for a clearer understanding of the natural history of CB across the lifespan. Such longitudinal studies have been scarce to date given the logistic challenges of maintaining them over time. In this review, we summarise current evidence on the evolution of the definitions, pathophysiology, risk factors, and consequences of childhood and adulthood chronic bronchitis.

## 1. Introduction

The term “bronchitis” was first used in the medical literature to describe “an inflammatory affection of the mucus membrane which lines the bronchial tubes” [1], with the first known recorded clinical case description dating back to the 17th century [2]. In 1932, Florey et al. published an illustration of the microscopic appearance of ciliated airway epithelial cells in chronic bronchitis (CB), showing goblet cell proliferation and hyperplasia, providing histological evidence of the mucous hypersecretion characteristic of CB [3].

The definition of CB was then refined by the British Medical Research Council (MRC) in 1959, using a standardised questionnaire on respiratory symptoms. Chronic cough (CC) was first defined as cough for more than three months; and chronic phlegm was defined similarly, and CB was then defined as a combination of having both CC and chronic phlegm for at least three months in two consecutive years [4]. These definitions have been widely used in studies and clinical practice [4,5]. However, this “traditional” definition of CB was initially used only to describe people with high exposures to tobacco smoking and ambient air pollution. This is no longer applicable for modern populations, including paediatric populations. 

Changes in definitions over time have likely led to a lack of comprehensive and consistent research into the burden of CC and/or CB in the population and their risk factors, which are still largely unknown [6,7]. Several terms have been used to describe “bronchitic” symptoms in the literature, such as productive cough, wet cough, and cough with phlegm. In general, this has been differentiated from CC without phlegm.

Nevertheless, as a key element of both CC and CB, cough itself is one of the most common reasons for patients seeking treatment in primary and secondary health care for both children and adults [8,9,10], and it has large impacts on quality of life (QoL). Children with cough had worse QoL [11] and were at increased risk of developing several chronic respiratory diseases (asthma and bronchiectasis), especially if wet cough persisted [12]. In adult studies, CC with phlegm was found to be associated with worsening QoL [13], impaired lung function [14], and increased all-cause mortality [15,16] independent of smoking. Although adult CB may be independent of airway obstruction, it has generally been viewed as a phenotype of chronic obstructive pulmonary disease (COPD)—the leading cause of respiratory morbidity and mortality worldwide—and indeed, in COPD, the prevalence of CB increases with the increasing severity of airway obstruction [17]. Thus, although not routinely confirmed using post-bronchodilator spirometry in primary care settings, CB with chronic airflow obstruction (“obstructive CB”) has a worse prognosis than CB without airflow obstruction (non-obstructive CB), as might perhaps be expected. However, both subtypes are predisposed to acute exacerbations, work absenteeism, and increased mortality [18].

In recent years, there has been substantial progress in finessing CC and CB in both children and adults in terms of their definitions, pathophysiologies, diagnosis, and treatment options. Given that cough is a key element in CB, this review first aims to describe the pathophysiology of cough and then aims to describe the evolution of the definitions, pathophysiology, prevalence, risk factors, and consequences of CB in children and adults (Table 1). 

## 2. Pathophysiology of Cough

An efficient cough means taking a deep breath in, closing the glottis tight, contracting the expiratory muscles against this to build up pressure behind the glottis, and then relaxing it suddenly to allow an explosive expiration with high flow rates, larger even than what can be achieved with a maximum expiratory breathing manoeuvre [19,20,21]. Coughing is normal, within limits; indeed, it is an important physiological mechanism to protect airways and lungs from inhaled particles and to remove excess mucus during airway infections, which may occur even normally several times per year. Cough is a reflex; there are vagal afferent nerve endings in the airway epithelium and within the lung tissue that send impulses to spinal cord ganglia. These messages are filtered in the medulla and midbrain and then, at a cortical level, they are modulated, either in a stimulatory or inhibitory manner [19,20]. Thus, we all cough, even when well, between 8 and 20 times per day [19]. When cough becomes more frequent and/or causes discomfort or “unease”, it becomes pathological. As an entity, cough may become an illness, or more frequently, it may be a manifestation of a provoking/sustaining underlying disease process. 

At its simplest, an efficient cough with sufficiently high expiratory flows will generate adequate shearing forces to remove particles or mucus from the airway epithelium surface. Even so, when flow rates are structurally low, as in moderate to severe COPD, cough will be inefficient, and this could be regarded as a common cause of cough pathology and a significant distressing contribution to the manifestation of “chronic bronchitis” in COPD. Here, there is a “double jeopardy”; excess mucus is produced, while the ability to eliminate it is reduced. Mucociliary clearance (MCC) is an important defensive mechanism in the airway to clear environmental pathogens. In this process, a protective mucous layer is secreted onto airway surfaces, and pathogens are trapped and mechanically removed through the mucociliary escalator using cilia and cough [22]. In CB patients, the three key elements of MCC efficiency, (i) airway hydration maintained by apical plasma membrane ion channels, (ii) ciliary beating, and (iii) mucin secretion, have all been found to be impaired, leading to the overproduction of mucus and an inability/inefficiency of cough to remove excess secretions [23]. Cough can be caused by exogenous factors (e.g., smoke, large particulates ~20 microns, cold air, etc.) that activate the afferent receptors responding to nociceptor C-fibres and/or A-delta fibres [19,20]; and endogenous factors (e.g., excessive airway mucus production and airway inflammation) that activate a range of specific epithelial- and lung-associated nerve ending receptors (chemical receptors) [19,20]. These chemical receptors encompass at least six sub-families of highly conserved Transient Receptor Potential (TRP) specific cation permeability channel receptors (including vanilloid, acid, and thermal modalities), a number of specific prostanoid receptors, adenosine triphosphate (ATP) receptors, tyrosine kinase receptors, histamine, and bradykinin receptors [19,20,24]. Prostanoids, particularly prostaglandin E (PGE2), emerge as key inflammatory mediators, but importantly, they potentially enhance cough responsiveness at both the levels of peripheral cough receptors and central cough receptors. Central neurological hypersensitivity in chronic cough involves PGE_2_ and the downregulation of gamma-aminobutyric acid (GABA)-mediated inhibitory mechanisms [25,26,27,28,29]. Capsaicin and histamine are notable cough inducers with transient receptor potential vanilloid (TRPV) receptor involvement [25], but they are not regarded as cough hypersensitivity inducers. ATP receptor antagonists have emerged as novel treatments of CC [30]. Targeting inflammatory mediators through medicines, such as the non-steroidal anti-inflammatory drug (NSAID) indomethacin, may offer therapeutic relief for cough hypersensitivity through anti-prostanoid effects. However, this proposition is currently based more on pharmacological theory than robust clinical trials, with limited evidence provided from a single small-scale randomised controlled trial [31,32]. This possibility warrants further study. Care needs to be taken in asthma as PGE_2_ may be important in stabilising the airway muscles to prevent excessive contraction, and there may also be diversion of arachidonic acid metabolism into leukotriene production, both effects leading to severe bronchospasm. Steroids are currently a better option than NSAIDs as anti-inflammatories in asthmatic cough at this stage, while new pharmacological strategies such as the use of ketamine [33], leukotriene modifiers [34], and theophylline are also being studied [35].

## 3. Evolution of Definitions and Pathophysiology of CB in Children

The hallmark symptom and sign of CB in children is protracted moist or wet/productive cough. Once more, the definition of chronicity varies. Australian and US guidelines define CC in children as >4 weeks [36,37], and UK guidelines define CC as >8 weeks [38]. Clinical criteria and nomenclature have evolved over time as mechanistic studies have advanced our understanding of the pathogenesis of CB in children. 

The definition of CB in children was refined in 2006 after Marchant et al. evaluated the cause of CC in 108 young children and showed that most had a clinical triad of (a) persistent wet cough, (b) a bacterial infection of the lower airways (found on bronchoscopy/broncho-alveolar lavage), and (c) a cough which resolved with 2 weeks of appropriate antibiotic therapy [39]. This syndrome of what was essentially CB was termed “Protracted Bacterial Bronchitis” (PBB) [39]. In 2010, the definition of PBB was refined further to increase its relevance to primary care and general paediatrics as bronchoscopy and broncho-alveolar lavage are not readily available in these settings. The revised “clinical definition” of PBB included (a) isolated chronic wet cough lasting >4 weeks, (b) resolution with appropriate antibiotic treatment, and (c) the absence of pointers indicating an alternative specific cause of cough [36,40]. This remains the most used definition and has been included in national and international cough guidelines [36,37,41]. Awareness of the importance of PBB, and its link to future bronchiectasis, has dramatically increased over time [12,42,43]. Whilst a range of possible aetiologies for this CB (i.e., PBB) are described, neutrophilic airway inflammation driven by bacterial infection of the airways is the most common local pathological mechanism in children [39,44]. Although the definition of CB essentially describes symptoms of PBB, a key element of the clinical definition of PBB, response to antibiotic treatment, is not a necessary condition to define CB. PBB is a better clinical definition as it provides clear-cut diagnostic criteria and treatment (i.e., antibiotics) [37,45].

The pathogenesis of PBB, as it is currently understood, is similar to bronchiectasis. There is also a condition called chronic suppurative lung disease (CSLD) in children, which may be a middle step between PBB and bronchiectasis. CSLD is used when a child presents with symptoms consistent with bronchiectasis, i.e., recurrent or prolonged episodes of chronic wet cough responsive to antibiotics, yet they have no radiological evidence of bronchiectasis. Therefore, PBB, CLSD, and bronchiectasis are considered part of a clinical spectrum with PBB at the mildest end and bronchiectasis rated the most severe, united by common clinical and microbiological features [45]. An initiating insult or insults (e.g., viral infection, bacterial pneumonia) impairs host pulmonary defences, resulting in airway bacterial overgrowth, mucous hypersecretion, and the impairment of mucociliary clearance [46]. The formation of epithelial surface biofilms in PBB promotes the persistence of bacteria, often non-typeable *H. Influenzae* (NTHi) or *M. catarrhalis*, in the airways [47], which likely contributes to the requirement for prolonged antibiotics. Environmental and genetic factors (both maternal and childhood factors) likely play an important role. For example, exposure to environmental irritants such as tobacco smoke and/or air pollution contributes to airway mucosal dysfunction that worsens airway inflammation, promoting bacterial overgrowth [48]. 

Whilst impairment in MCC likely plays an important role in PBB pathogenesis, aberrations in the innate immune system have also been implicated. For example, elevated levels of interleukin (IL)-8, active-matrix metalloproteinase-9, and IL-1β have been shown in several studies [49,50,51], implicating the neutrophil inflammatory response in PBB pathogenesis. Further, impaired apoptosis and efferocytosis to NTHi [51] and altered gene expression in NTHi-stimulated lower airway cells [52] have been found in children with PBB compared to controls and those with bronchiectasis.

Certain populations, e.g., Aboriginal and Torres Strait Islander children in Australia, Māori and Pacific Islanders in New Zealand, and Alaskan children in the United States, are known to be at an increased risk of CB and bronchiectasis, and efforts are currently underway to understand the specific mechanisms underpinning this increased susceptibility [53,54,55].

There is a diagnostic and management algorithm specifically recommended for children with chronic cough (including those with PBB) which is based on high-quality evidence from systematic reviews and randomised controlled trials [6,36,37]. Importantly, indicators of serious pathology related to cough in children (e.g., dyspnoea, chest pain, abnormal chest X-ray, etc.) should be assessed with further investigations and monitoring as needed for children who are at a high risk of developing bronchiectasis specifically [6,36,37]. For PBB, antibiotics are usually the first-line treatment [37,45,46], and there is a need to develop novel therapeutics for recurrent airway exacerbations [45].

## 4. Evolution of Definitions of CB in Adults

As can be gleaned from the above discussion, the semantics and characterisation of cough and related syndromes in adults over time have been complex, leading to confusion, and difficulties remain for the current nomenclature. The story of modern definitions probably starts with attempts by the UK MRC to come to terms with the epidemiology and community impacts of severe air pollution in the 1950s, accompanied by an accelerated uptake of cigarette smoking during the Second World War. In 1959, MRC researchers developed an empiric and arbitrary definition of what was termed “chronic bronchitis” as productive cough (with sputum) for three months of each year over two consecutive years [4]. At much the same time, pathologists such as Lynne Reid (in London) were examining smokers’ airways. She described a remodelling of the airway epithelium with a loss of normal cellular pseudo-stratification with squamous metaplasia accompanied by hyperplasia of mucus-secreting goblet cells and hypertrophy of sub-epithelial mucous glands [56]. Not unreasonably, these pathological changes were taken as correlates of the disease of “epidemiological” CB coined by the MRC. Subsequently, this was adopted as a clinical diagnosis. Further, many smokers went on to develop chronic airflow obstruction, termed COPD, clinically. CB was then included as the third pillar of a full-blown picture of COPD. However, with time, the picture proved to be rather more complicated; many individuals with CC did not have accompanying sputum and/or did not fit the usual three-winter-months-of-cough paradigm (the cool and wet winter months in UK, from the MRC’s perspective). In addition, overall, there was a poor correlation between smoking, airflow obstruction, and cough. However, the label of CB had been absorbed into respiratory clinical culture and has been hard to eradicate. Its current clinical use encompasses far more than the early definitions and presumptions suggested.

Currently CB in adults is still defined as cough with phlegm for more than three months in two consecutive years using the MRC questionnaire, but many attempts have been made to refine the definition of CB to address the above limitations. The aim is to develop better symptom profiles and a better understanding of how these profiles relate to acute and chronic illness patterns and physiological changes. A recent study identified six distinct cough subclasses based on cough and phlegm symptoms derived from the MRC questionnaire [14]. The two most clinically relevant cough subclasses, namely “chronic productive cough” and “intermittent productive cough”, had similar clinical features to the MRC definition of CB but were distinctively different from the “chronic dry cough” subclass [14]. 

## 5. CB in Children: Prevalence, Risk Factors, and Consequences

The prevalence of CB in the general paediatric population is largely unknown [40] as existing studies have focused on the prevalence of PBB or chronic wet cough among children presenting to different healthcare facilities [39,57,58,59,60]. As stated in the European Respiratory Society’s PBB task force document, the prevalence of PBB in community settings is a major research gap that should be addressed using large population-based studies [40].

The only study that recruited children from a community followed 203 Australian Aboriginal children aged under 7 years from four remote communities and found the prevalences of chronic wet cough and PBB were 13% and 10%, respectively [61]. 

The prevalence of CB in children varies by setting. An Australian multi-centre study including eight hospitals recruited 346 children under the age of 18 years who presented with chronic cough. The study found that the prevalence of PBB among children with chronic cough varied significantly across different hospitals, ranging from 16% to 54% [59]. Another study found that only 12% of the 156 children presenting to a tertiary hospital with chronic cough (aged 5–16 years) had PBB [57]. Two further studies have reported prevalences of both chronic wet cough and PBB among children presenting to tertiary hospitals. The first found that among 171 children with persistent cough, 59 (34.5%) had chronic wet cough, and 47% (55 out of 117) of children who had persistent cough and were reviewed by a paediatric pulmonologist had PBB [58]. The other study found that among 108 children with chronic cough, 89% had wet cough and 40% had PBB [39].

There are very few studies assessing risk factors specifically for CB in children. One study found that among high school students (mean age 17 years), e-cigarette smoking increased the risk of having CB symptoms [62]. Another found that premature birth (ranging from 32 to 37 weeks) increased the risk of persistent chronic wet cough in children under 3 years [63]. A few studies assessed risk factors for CC in children without the specification of the presence of phlegm (or “chronic bronchitis”) and found that outdoor air pollution [64], childcare attendance [65], and homelessness [66] all increased the risks of having unspecified CC during childhood. As for the aetiology of CC in children, PBB is the most common cause of wet cough, followed by bronchiectasis, and asthma. For unspecified CC [67,68], dry cough is more likely to be caused by croup, tracheobronchomalacia, psychogenic cough, and vascular and airway abnormalities [69]. 

The natural history of CB is largely unknown as there are very few prospective studies which follow bronchitis from childhood to adulthood. One study followed children with PBB (aged < 14 years) prospectively at 1 [42] and 5 years after their initial diagnosis [12] to assess disease prognosis and potential risk factors. It found that 8.1% of children with PBB developed bronchiectasis at 1 year, increasing to 9.6% at 5 years [12,42]. Both recurrent episodes of PBB and Haemophilus influenza infection were significant predictors of bronchiectasis [12,42]. Additionally, 27.1% of children with PBB were diagnosed with new-onset asthma at 5 years. Developing asthma after PBB was found to be associated with bronchomalacia and positive allergy-specific IgE at baseline [12,42]. However, the impact of childhood PBB on adulthood respiratory health is still unknown as the median age of the participants at the 5-year follow-up was only 8 years [12].

In the population-based Tasmanian Longitudinal Health Study (TAHS), which prospectively followed participants from age 7 to 53 years, CB was repeatedly measured (reported by parents in childhood or self-reported in adulthood) [70]. It found that childhood bronchitis was associated with doctor-diagnosed current asthma at age 43 years of age [71], ever had pneumoniaby age 43 years [71], longitudinal asthma phenotypes from childhood to middle-age [72], lifetime obstructive and mixed (both obstructive and restrictive) spirometry patterns [73], rapidly declining FEV_1_ and FEV_1_/FVC values and trajectories [74], as well as COPD at 53 years of age [75]. Childhood chronic bronchitis was also negatively associated with asthma remission by middle age [76]. As the TAHS collected self-reported/parent-reported respiratory symptoms to define “bronchitis” in 1968 and did not specify that these symptoms occurred independently of wheeze, the results may not be entirely applicable to the current definitions of PBB in children. The use of “recurrent and protracted attacks of bronchitis” or “loose or rattly” cough in the THAS was probably the nearest to reality (i.e., PBB) when the data were collected in 1968 [71].

## 6. CB in Adults: Prevalence, Risk Factors, and Consequences 

The prevalence of CB symptoms varies widely across general adult populations worldwide but is around 3–6% in Westernised countries [77,78,79,80,81,82]. It is more common in men [77] and increases with advancing age, tobacco smoking, occupational exposures [83,84], obstructive sleep apnoea [85], and lower socioeconomic status [78]. However, adults of the general population who experience symptoms of chronic bronchitis often do not have a formal diagnosis [78], which would underestimate its true prevalence. This underdiagnosis is likely to be in part due to the common label of a “smoker’s cough”, which may discourage affected adults from presenting to primary care and deter health care professionals from arranging post-bronchodilator spirometry to confirm complications such as COPD and obstructive CB [86]. CB is more common in men and persons of lower socioeconomic status [78].

Of the known risk factors associated with adult CB, the most important causative factor is personal exposure to cigarette smoke due to active smoking and/or passive inhalation. Having a productive cough from personal smoking is more prevalent in people with a greater daily smoking intensity and higher cumulative pack-year history [87]. Around one-third of middle-aged patients in psychiatric hospitals report CB symptoms [88], which reflects the high smoking rates in this population (43–88%). In addition to tobacco, there is also evidence suggesting associations between vaping/e-cigarettes, cannabis, and chronic bronchitis [36].

Among those susceptible to infection, repeated “colds that go to the chest” and other lower respiratory tract infections often precede chronic bronchitic symptoms, especially those relating to non-typable strains of Haemophilus influenzae (NTHi) [89]. Those who are socially disadvantaged have increased vulnerability to recurrent chest infections as they tend to live in overcrowded housing with limited heating, are undernourished, smoke tobacco, and have reduced access to primary health care [90]. 

Symptoms of CB overlap with those of other obstructive lung diseases such as cystic fibrosis, bronchiectasis, and asthma [7], which are important differential diagnoses for productive cough. In the TAHS study, concurrent asthma and/or wheezing in middle-age was also found to be independently associated with adult CB (odds ratio [OR]: 6.2 [95% CI: 4.6, 8.4]), and this estimate was stronger than for participants who currently smoked with a ≥20 pack-year history (OR: 3.0 [95% CI: 2.1, 4.3]) [79]. However, the associations of smoking and current asthma in relation to obstructive CB were strong, while allergy was associated with non-obstructive CB in middle age [79]. This overlap of symptoms of productive cough in active asthma may feasibly relate to mucoid rather than purulent sputum, but this was not formally studied. 

A lower income and occupational category can also predispose to CB in adults, with the highest prevalence seen in manual workers and self-employed individuals [77,78]. A review conducted by the American Thoracic Society and European Respiratory Society on the contribution of workplace exposures to lung diseases estimated that the population-attributable fraction of the CB burden from occupational factors was 13% (95%CI: 6, 21) [91]. The strongest drivers of this burden were exposure to metals and mineral dusts [80,92]; the prevalence of CB among coal miners increased with job duration and cumulative dust exposure [93]. Among farmers, positive associations between different livestock trades and CB have varied according to whether farmers work with dairy cattle, swine, and horses [94,95]. Pesticide use, which includes exposure to insecticides, fungicides, and herbicides, has been consistently linked to adult CB [96].

Ambient air pollutants from traffic and industrial sources have the potential to irritate the airways and trigger cough and phlegm symptoms [97], but findings from studies vary with significant heterogeneity in study design, preventing a pooled estimation to synthesise the evidence [7]. Similarly, non-cohort studies showing associations between an increased risk of CB and household air pollution from biomass fuel burning in poorly ventilated areas are typically limited to women from low-to-middle-income countries [98]. Epidemiological data on the impact of wildfire smoke exposure are also lacking [99].

In the TAHS study, childhood factors, which included allergies (a food or medicine allergy, childhood eczema, and/or hives), asthma (symptoms in the last 12 months or “episodic asthma” lasting 1–4 weeks every 3 months or lasting 1 week every 2–4 weeks), and bronchitic symptoms in one or both parents when their offspring were children, were found to be associated with CB and airway obstruction at an age of 43 years [79,100]. Asthma was a risk factor of CB, as was an FEV1 < 80%, as predicted. This was also supported by the Tucson Epidemiologic Study of Airway Obstructive Diseases, in which “active” physician-diagnosed asthma was associated with a 10-times-higher risk of acquiring CB in early adulthood [95%CI: 4.9, 20.2] [101]. The TAHS study also found that people with CB or chronic productive cough subclasses at an age of 53 years had a significantly higher prevalence of CB in earlier life (from age 7 to 43 years) [14].

Thus, the current evidence from population-based studies reinforces the concept that CB can arise from factors other than the major causative risk factor, cigarette smoking. Taken together, the independent associations of childhood allergy and asthma as well as childhood bronchitis suggest that some of the disease burden of adult CB also originates in childhood.

As for the treatment and management of adult CB patients, evidence is limited. Current international guidelines on chronic cough have recommended treating cough as per its related aetiologies (e.g., asthma, GORD, etc.) and/or non-specific antitussive treatments (i.e., potentially using neuromodulators and ATP antagonists for cough hypersensitivity; ICS for eosinophilic inflammation) [36,41,102,103] rather than basing it on the presence of phlegm (i.e., dry cough or productive cough/chronic bronchitis). This is probably because earlier studies have shown that the presence of phlegm provides little value to indicate the potential aetiologies of a cough [9,102,104]. In addition to treating for related conditions (e.g., COPD, asthma, etc.) and smoking cessation for smokers with CB, there are some CB-specific treatments, including mucolytics and macrolide antibiotics, but more evidence is needed before recommendations can be made [36]. 

## 7. Conclusions

The definition of CB for both adults and children has evolved over the years, and the emerging novel clinical entities of CC, PBB, cough hypersensitivity, and other novel cough subclasses may be better classifications of cough-related conditions in the context of the diagnosis and management of patients, and even in epidemiological research. Further, chronic productive/wet cough in both children and adults may require medical attention and monitoring, considering its links with future respiratory health and all-cause mortality. Detailed clinical work-up is recommended for both children and adults with prolonged bronchitic symptoms (chronic productive cough), and regular follow-ups, especially including spirometry, may be required. 

The current evidence on prevalence is highly heterogenous across different population groups and partly related to the use of different definitions in studies. In both children and adults, environmental stimuli such as smoke, air pollution, and infection can trigger bronchitic symptoms, but more studies are needed to understand other potential modifiable risk factors for CB. Understanding the true burden of CB (and similar conditions such as PBB, CC, wet cough, productive cough, etc.) and its risk factors is currently a major research gap. 

CB in childhood may persist for years and even decades and is associated with the future development of asthma, bronchiectasis, and lung function impairment. However, childhood asthma and allergies are predictors of adulthood CB in middle age. The interplay between risk factors, childhood CB, and adulthood respiratory health (including CB) is complicated. Future studies should focus on the natural history of CC and CB separately over the life course using data from high-quality, large-scale longitudinal cohorts to further disentangle the most relevant associations.

## Figures and Tables

**Table 1:** Contrasting characteristics and/or nomenclature of “chronic bronchitis” in children versus adults.

Characteristics		Children	Adults
**Current definition**		Protracted bacterial bronchitis (PBB) is the most clinically relevant definition for children, defined as a daily wet cough lasting >4 weeks, resolution with appropriate antibiotics (usually amoxicillin-clavulunate), and an absence of indicators to suggest an alternative cause for cough. Other definitions include wet cough and chronic cough.	Chronic bronchitis: cough with phlegm for 3 months or more in 2 consecutive years. This may no longer be as applicable to the modern population as when formulated and novel definitions are emerging, especially for cough hypersensitivity.
**Risk factors**	Common	Infection, indoor and outdoor air pollution, tobacco/e-cigarette/cannabis smoking (active and passive)	
	Socio-economic	Childcare attendance; homelessness; household crowding	Lower income and some occupations
	Others	Premature birth	Childhood asthma, allergies, cough, OSA
**Consequences**		Bronchiectasis; asthma; impaired lung function including COPD; reduced quality of life	
**Management**		Antibiotics	Treating related conditions (e.g., asthma, GORD); smoking cessation; mucolytics; macrolide antibiotics

COPD: chronic obstructive pulmonary disease; GORD: gastro-oesophageal reflux disease; OSA: obstructive sleep apnoea.
